# The influence of rising carbon dioxide on maize development: genotypic differences in growth, lignification and folate pathway

**DOI:** 10.1002/jsfa.70251

**Published:** 2025-10-16

**Authors:** Pirzada Khan, Sajjad Asaf, Lubna, Eman R Elsharkawy, Rahmatullah Jan, Kyung Min Kim

**Affiliations:** ^1^ Biotechnology Research Institute, Chinese Academy of Agriculture Sciences Beijing China; ^2^ Natural and Medical Science Research Center University of Nizwa Nizwa Oman; ^3^ Center for Health Research Northern Border University Arar Saudi Arabia; ^4^ Coastal Agriculture Research Institute, Kyungpook National University Daegu Republic of Korea; ^5^ Department of Applied Biosciences Kyungpook National University Daegu Republic of Korea

**Keywords:** carotenoids, elevated CO_2_, folate metabolism, lignin biosynthesis, maize growth

## Abstract

**BACKGROUND:**

Rising atmospheric carbon dioxide (CO_2_) is a key driver of climate change, making it essential to understand its effects on crop growth and metabolism. This study examines maize C01 (inbred) and B73 (mutant), under elevated CO_2_ (600, 1200 and 1800 ppm) at three growth stages [40, 70 and 90 days after sowing (DAS)].

**RESULTS:**

At 600 ppm CO_2_, plant height, leaf area and biomass increased, whereas higher concentrations led to significant declines. C01 accumulated more sugars and total non‐structural carbohydrates, whereas B73 showed higher starch levels. Chlorophyll and carotenoid contents decreased under elevated CO_2_, with the most pronounced reductions at 1800 ppm. Folate content peaked at 70 DAS, with B73 exhibiting consistently higher levels than C01. Lignin accumulation and composition varied across genotypes, tissues and CO_2_ levels. At 600 ppm, lignin content increased in leaves and stems but declined at 1800 ppm. The syringyl‐to‐guaiacyl ratio and lignin monomer composition differed between genotypes, with C01 displaying stronger phloroglucinol staining and higher lignin content than B73. Gene expression analysis revealed that key lignin biosynthesis genes (*ZmPAL*, *ZmCAD*, *ZmCCR*, *Zm4CL*, *ZmCOMT* and *ZmCCoAOMT*) were upregulated at 600 ppm but significantly downregulated at 1800 ppm, particularly in B73.

**CONCLUSION:**

These findings highlight genotype‐specific responses to elevated CO_2_, emphasizing its influence on maize growth, lignin biosynthesis and folate metabolism. The study provides valuable insights for future crop management and breeding strategies in the face of rising atmospheric CO_2_ levels. © 2025 The Author(s). *Journal of the Science of Food and Agriculture* published by John Wiley & Sons Ltd on behalf of Society of Chemical Industry.

## INTRODUCTION

The rising concentration of atmospheric carbon dioxide (CO_2_) is a primary driver of global warming and climate change, with levels increasing from 280 ppm before the industrial revolution to 409.23 ppm in 2018, and projections of 800 ppm by 2100.^1^, ^2^, ^3^ This escalation significantly influences agriculture, affecting plant physiology, photosynthesis and crop productivity.^4^ Elevated CO_2_ enhances carbon assimilation, energy storage and biomass production,^5^, ^6^ and also increases photosynthesis and alters metabolite production in C_4_ crops such as maize (*Zea mays* L.).^7^ However, elevated CO_2_ can reduce phytohormone and phytochemical production, potentially weakening plant defenses against biotic stresses.^8^ Maize, a globally significant C_4_ crop, is crucial for food, ethanol and starch production, but is highly susceptible to climate instability, with regions such as southern Brazil already experiencing yield reductions.^9^ Understanding maize's response to elevated CO_2_ is critical for mitigating climate change impacts and ensuring food security.

Elevated CO_2_ functions as both a substrate and a signal, enhancing photosynthesis at the same time as affecting nutrient dynamics. This ‘CO_2_ fertilization effect’ has been shown to improve crop productivity in C_3_ plants by 20–30% under controlled environments such as growth chambers, open‐top chambers (OTCs) and free‐air CO_2_ enrichment (i.e. FACE) facilities.^10^, ^11^ Elevated CO_2_ significantly lowers essential nutrients like nitrogen, phosphorus, potassium, and magnesium by 5–25%, thereby reducing the nutritional quality of crops and exacerbating malnutrition risks.^12^, ^13^ These nutrient imbalances have far‐reaching implications for soil nutrient cycles and human health.^14^

Folate plays a central role in metabolic processes, including nucleic acid and amino acid synthesis, and is critical for plant development, nitrogen metabolism and stress responses.^15^, ^16^ Biofortification efforts in staple crops such as rice, tomato and lettuce have achieved some success, but challenges remain because of species‐specific pathway regulation and folate instability during storage.^17^, ^18^ Understanding folate metabolism in plants under elevated CO_2_ is essential for developing biofortified crops with enhanced nutritional quality and stress tolerance.

Lignin, a phenylpropanoid polymer crucial for plant structural integrity, water transport and defense, constitutes approximately 30% of organic carbon in the biosphere.^19^ Elevated CO_2_ often increases lignin synthesis by stimulating the phenylpropanoid pathway, although responses vary depending on species, genotype and nutrient availability.^20^ Transcriptomic analyses in crops such as *Arabidopsis* and soybean reveal upregulation of lignin‐related genes under elevated CO_2_, including *PAL1*, *LAC4*, *COMT* and *CCR*, whereas studies in *Populus tremuloides* demonstrate genotype‐dependent variations in lignin biosynthesis and carbon partitioning.^21^, ^22^ However, limited data on quantitative lignin content under elevated CO_2_ necessitates further investigation into its role in structural development and biochemistry.

Long‐term exposure to elevated CO_2_ can downregulate photosynthesis by reducing RuBisCO activity, altering nutrient dynamics, and increasing tissue C:N ratios, with responses strongly influenced by light and nutrient availability.^23^, ^24^ Moreover, species differ in their sensitivity because herbaceous plants often show reduced nitrogen content and smaller photosynthetic gains compared to woody species.^25^ However, there is a lack of studies investigating the effects of elevated CO_2_ on lignin and its derivatives, chlorophyll, carotenoids, folate and soluble sugars in maize. Therefore, the present study comprehensively examined the physiological and biochemical responses of maize to elevated CO_2_, with a particular emphasis on folate biosynthesis and lignin accumulation. By integrating molecular, biochemical and physiological analyses, the research seeks to advance understanding of maize adaptation to future climate scenarios and provide insights for breeding strategies aimed at enhancing crop resilience, nutritional quality and sustainability.

## MATERIALS AND METHODS

### Experimental site

An experiment utilizing OTCs was conducted at the Langfang research farm (39.5380°N, 116.6838°E) in Hebei Province, China, characterized by a cold, temperate climate. The site experiences a mean annual temperature of 12.1 °C and an average annual precipitation of 557 mm. The frost‐free period typically ranges from 180 to 200 days per year. Precipitation is unevenly distributed, with approximately 75% occurring in the summer, and rainstorms are most frequent in July and August.

### Experimental design and elevated CO_2_ treatments

The effects of elevated CO_2_ on the agronomic and biochemical characteristics of maize (*Z. mays* L.) were evaluated through chamber experiments. In response to current atmospheric CO_2_, maize seeds were cultivated under three CO_2_ levels: 600, 1200 and 1800 ± 20 ppm. The experiments took place during the Kharif season (mid‐August to mid‐November) in 2018 and 2019, using OTCs (Table 1). Plants were spaced 20 cm apart within rows, with 60 cm between rows. Elevated CO_2_ levels in the OTCs were maintained between 08.30 h and 18.00 h by releasing CO_2_ gas from 30‐kg capacity cylinders positioned around the chamber perimeter, 35 cm above the soil surface. Air blowers located at the chamber base ensured adequate mixing of CO_2_ with incoming air. A CO_2_ analyzer, regulated by a computerized system, continuously monitored and adjusted CO_2_ concentrations via inlet valves. CO_2_ was supplied during daylight hours to support photosynthesis because plants do not respond to elevated CO_2_ levels in the absence of light. Crops received fertilizer at rates of 120 kg of nitrogen, 26 kg of phosphorous and 50 kg of potassium per hectare. Phosphorus, potassium and half of the nitrogen were applied at sowing. The remaining nitrogen was split into two equal applications: the knee‐high stage [25 days after sowing (DAS)] and the tassel formation stage (65 DAS).

**Table 1:** Experimental treatments

Treatment no.	Name	Description
T1	C1	Elevated CO_2_ (600 ± 20 ppm)
T2	C2	Elevated CO_2_ (1200 ± 20 ppm)
T3	C3	Elevated CO_2_ (1800 ± 20 ppm)

### Open top chamber design

**Figure 1:**
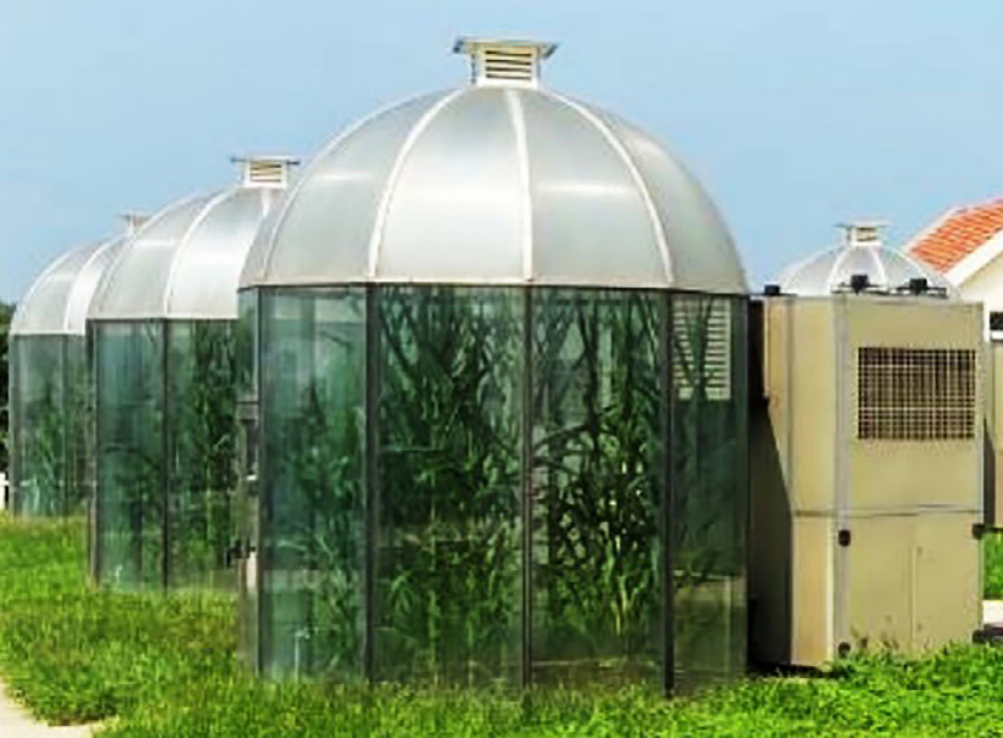
Open top chamber used in the present study.

Each open‐top chamber (OTC) in the four pairs was constructed with a height of 3.5 m and an octagonal base covering a surface area of 3.73 m^2^ (Fig. 1). To prevent mutual shading effects, a buffer zone 5.5 m wide was maintained between adjacent chambers. CO_2_ gas, sourced from liquid CO_2_ cylinders (purity: 99.99%) provided by Anjin Gas Corporation (Langfang City, Hebei Province, China), was delivered to the chambers through perforated pipes positioned outside the structures. Within each OTC, a fan facilitated the mixing of the supplied CO_2_ with fresh air from outside the chamber, ensuring uniform distribution throughout the enclosure. The CO_2_ exited through the top opening, promoting ventilation and the exchange of air. The concentration of CO_2_ inside the chambers was continuously monitored using an infrared gas analyzer to maintain accurate levels.

### Growth and harvesting of maize

In this study, the impact of elevated CO_2_ on *Z. mays* was evaluated across the growing season. Two maize lines were used: the inbred line C01 (wild‐type) and an EMS‐induced B73 (mutant, F_3_ generation) line capable of accumulating 5‐methyltetrahydrofolate. Plants were harvested at three developmental stages: the vegetative stage (40 DAS), tasseling stage (70 DAS) and dent stage (90 DAS). Various morphological and biochemical parameters were assessed at each stage. Plant height, leaf area and the fresh and dry weights of leaves and stems were recorded. Leaf area was calculated using the length‐width coefficient method (length × width × 0.75), as described previously.^26^ Total leaf area per plant was determined by summing the individual leaf areas. Aboveground biomass was quantified by measuring the dry weight of leaves and stems. For each treatment, six plants were sampled and separated into leaves and stems. The plant parts were dried in a forced‐air oven at 60 °C for at least 96 h until a constant weight was achieved. The dry weights of leaves and stems were then measured using an electronic balance.

### Measurement of chlorophyll content

To determine the chlorophyll content, 300 mg of fresh leaf tissue was weighed and placed in a 15‐mL Falcon tube. The sample was then extracted using 80% acetone, ensuring thorough mixing in the dark for 15–30 min. Following extraction, the samples were centrifuged at 3000 x g for 15 min at 4 °C. The supernatant was mixed thoroughly and the absorbance (*A*) of the chlorophyll extract was measured using a spectrophotometer. Absorbance readings were taken at 663 and 645 nm, with 80% acetone serving as the blank control. Chlorophyll concentrations were calculated using^27^:

$$\text{Chlorophyll}\;a\;\left(\mathrm{Ca},\mathrm{mg}\;g^{–1}\right)=\left[\left(12.7\times A_{663}\right)–\left(2.69\times A_{645}\right)\right]\times V/\left(1000\times W\right)$$

$$\text{Chlorophyll}\;b\left(\mathrm{Cb},\mathrm{mg}\;g^{–1}\right)=\begin{array}{l} \left[\left(22.9\;\times \;A_{645}\right)–\left(4.86\;\times \;A_{663}\right)\right]\\ \;\times \;V/\left(1000\;\times \;W\right) \end{array}$$

where *V* is the volume of the extract (mL) and *W* is the weight of fresh leaf tissue (g)

For carotenoid quantification, the same extract was used, and absorbance was measured at 470 nm. Carotenoid contents was calculated using:

$$\text{Carotenoid}=\left\{\left[\left(1000\times A_{470}\right)–\left(1.82\times \mathrm{Ca}\right)–\left(85.02\times \mathrm{Cb}\right)\right]/198\right\}\;\times \;V/\left(1000\times W\right)$$

These calculations provided the chlorophyll *a*, chlorophyll *b* and carotenoid per gram of fresh leaf tissue.

### Total soluble sugar and starch contents

Total soluble sugar and starch contents were quantified using commercially available chemical kits (Solarbio Life Sciences, Beijing, China) in accordance with the manufacturer's instructions. Briefly, 200 mg of fresh leaf tissue was placed in a 1.5‐mL tube and 1 mL of distilled water was added. The mixture was vortexed thoroughly and incubated for 10 min. After cooling, the sample was centrifuged at 3000 × *g* for starch extraction and at 8000 × *g* for sugar extraction at room temperature for 10 min. The resulting supernatant was filtered and transferred to a 10‐mL test tube, then diluted to a final volume of 10 mL with distilled water. For quantification, glucose was used to prepare the standard curve. A 200‐μL aliquot of the sample extract was mixed with anthrone reagent and 1 mL of 95% sulfuric acid. The mixture was heated in a water bath at 95 °C for 10 min. After cooling, the absorbance was measured at 620 nm using a UV‐1200 spectrophotometer.

### Total non‐structural carbohydrate (TNC)

The TNC content was determined by summing the measured amounts of total soluble sugar (TSS) and starch. This approach follows the methodology described previously,^28^ where non‐structural carbohydrates are quantified to assess the readily available energy reserves within plant tissues. Specifically, TSS represents the pool of free sugars, such as glucose, fructose and sucrose, whereas starch reflects the primary storage form of carbohydrates. Combining these two components provides a comprehensive measure of the plant's energy reserves, which are critical for understanding its growth, metabolism and response to environmental conditions.

### Folate extraction

Folate extraction was carried out following the method described previously,^29^ with slight modifications. Fresh leaf tissues were finely ground into a powder using a mortar and pestle under liquid nitrogen. From this, 50 mg of the powdered tissue was transferred into a 1.5‐mL screw‐cap tube (ST‐150; Axygen, Union City, CA, USA). The sample was mixed with 1 mL of freshly prepared extraction buffer, consisting of 50 mm phosphate buffer (pH 7.0), 0.5% sodium ascorbate and 0.2% *β*‐mercaptoethanol, until a homogeneous mixture was achieved. Methotrexate was added to the extraction buffer as an internal standard before mixing. The mixture was boiled in a water bath for 10 min and subsequently cooled on ice for 10 min. To facilitate the deconjugation of folate polyglutamates, 30 μL of rat serum was added to each sample, followed by incubation at 37 °C for 4 h. The sample was then boiled for another 10 min, cooled on ice for 10 min, and centrifuged at 13,000 x g at 4 °C for 10 min. After centrifugation, 400 μL of the supernatant was transferred to a 3‐kDa ultrafiltration tube (Millipore, Burlington, MA, USA) and centrifuged again at 13,000 x g at 4 °C for 20 min. The resulting filtrate was collected for folate analysis. Each sample was processed in triplicate to ensure accuracy and reproducibility of the results.

### Extraction and quantification of folate derivatives evaluation

The folate derivatives extraction procedure was then performed. Pre‐cooling solutions of 80% methanol in water and methanol containing 15 mg/mL dithiothreitol (DTT) were prepared in advance and stored at −20 °C. Additionally, isopropanol was also pre‐cooled to −20 °C. Approximately 100 mg of fresh leaf tissue was ground into a fine powder, and 300 μL of pre‐chilled 80% methanol aqueous solution was added. To this, 100 μL of the pre‐prepared methanol‐DTT solution (15 mg mL^−1^) was added. The mixture was vortexed and shaken for 1 min to ensure thorough mixing. The sample was then placed on ice for 30 min and centrifuged at 12,000 x g for 10 min at 4 °C. The supernatant was carefully transferred to a new centrifuge tube, and 300 μL of pre‐chilled isopropanol along with 100 μL of the methanol‐DTT solution was added to perform secondary extraction. The mixture was vortexed and shaken for 1 min, then placed on ice for an additional 30 min before being centrifuged again at 12,000 x g for 10 min at 4 °C. The supernatant from this second centrifugation was combined with the supernatant from the first extraction. The combined supernatant was filtered using a 0.22 μm organic membrane filter (YA0699; Sol–Gel, Golda Meir 7, Ness Ziona, Israel) to remove any remaining particulate matter. The filtered extract was stored at −20 °C for subsequent analysis using liquid chromatography‐mass spectrometry. This method allows for efficient extraction and preparation of the sample for accurate folate quantification.

### Lignin contents and composition

Lignin was extracted in the midrib and stem of C01 and B73 by the acetyl bromide method.^30^ using the commercially available chemical kit in accordance with the manufacturer's instructions (Solarbio Life Sciences, Beijing, China), with a 3 mg sample being used after passing through a 40 mesh sieve into a 1.5‐mL EP tube. The sample was mixed with perchloric acid acetylated in an 80 °C water bath for 40 min. After cooling the samples were centrifuged at 8000 x *g* for 10 min, the supernatant was collected and mixed with glacial acetic acid using a spectrophotometer (UV‐1200 MAPADA, Shanghai Mapada Instruments Co., Ltd, Shanghai, China) at an absorption of 280 nm. Thioacidolysis and gas chromatography–mass spectrometry (GC–MS) were used to determine lignin composition according to a previously reported method.^31^ The whole leaf and stems were harvested at three different stages until completely dry. The collected samples were ground in a grinder until they complete powder. The dry powder was used for lignin composition analysis after passing through a 40‐mesh sieve. H, G and S lignin were identified and quantified by GC–MS using a model 5890 series II gas chromatograph (Hewlett‐Packard, Palo Alto, CA, USA) with a 5971 series mass selective detector (column HP‐1, 60 m 9 0.25 mm, film thickness 0.25 μm; Agilant, Santa Clara, CA, USA).

### Phloroglucinol staining and microscopic analysis of lignin in maize midrib sections

Midribs from C01 and B73 maize plants at 40, 70 and 90 days of growth were sectioned using a microtome machine (Wilkinson Sword, London, UK) and stored in sterile distilled water for no longer than 2 h until all sample sectioning was completed. Phloroglucinol staining was performed with modifications based on the method described previously.^32^ A 1% stock solution of phloroglucinol (Sigma‐Aldrich, St Louis, MO, USA) was prepared by dissolving the compound in 95% ethanol (Decon Laboratories Inc., King of Prussia, PA, USA). Immediately before use, concentrated hydrochloric acid (33% v/v) was mixed with the stock solution to prepare the phloroglucinol staining solution. The maize tissue samples were placed on glass slides (Thermo Fisher Scientific, Waltham, MA, USA) and excess solution was removed using Kimwipes (Kimberly Clark, Irving, TX, USA). Phloroglucinol stain (3 μL) was applied to the tissue for 30 s, followed by the application of a cover glass (Corning Inc., Corning, NY, USA). Additional phloroglucinol staining solution was added to ensure that the sample was completely covered. Light microscopy images were captured using a Spot RT Slider camera (Diagnostic Instruments Inc., Sterling Heights, MI, USA) mounted on a Nikon Eclipse E800 microscope (Nikon, Tokyo, Japan) at 20× magnification. Image analysis was performed using SPOT, version 4.0.6 software (Diagnostic Instruments Inc.).

### RNA extraction and quantitative PCR

RNA extraction was performed using the EasyPure® Plant RNA Kit (ER 301‐01; All Gold; TransGen Biotech, Beijing, China). The extracted RNA was stored at −80 °C or immediately used for reverse transcription. RNA concentration was quantified using a NanoDrop One/OneC spectrophotometer (Thermo Fisher Scientific). For cDNA synthesis, 1 μg of total RNA was reverse transcribed using the RevertAid First Strand cDNA Synthesis Kit (K1622; Thermo Fisher Scientific) in accordance with the manufacturer's instructions. Quantitative PCR (qPCR) for *ZmPAL*, *Zm4CL*, *ZmCAD*, *ZmCOMT*, *ZmCCR* and *ZmCCoAOMT* was carried out using Top Green qPCR Supermix (+Dye II) (AQ 132‐21; TransGen Biotech). The primer sequences and corresponding gene accession numbers are listed in the Supporting information (Table S1). The reaction was set according to the method described previously.^33^ Briefly, the amplification conditions consisted of an initial denaturation at 95 °C for 2 min, followed by 40 cycles of 94 °C for 10 s, 60 °C for 10 s and 72 °C for 40 s. Actin was employed as the reference gene, with all reactions performed in three technical replicates. To ensure the stability of actin expression, its transcript levels were evaluated under all experimental treatments using three independent biological replicates. Relative gene expression was determined using the 2^−ΔΔCt^ method.

### Statistical analysis

Two‐way analysis of variance was performed to analyze the impacts of elevated CO_2_ on maize seedlings using Statistics 10 (Analytical Software, Tallahassee, FL, USA). A least significant difference test was performed for mean comparison at a 5% level of significance. Prism, version 7.0 (GraphPad Software Inc., San Diego, CA, USA) was used for image construction. Data are expressed as the mean ± SE of triplicates.

## RESULTS

### Impact of elevated CO_2_ on maize morphology and growth dynamics

**Figure 2:**
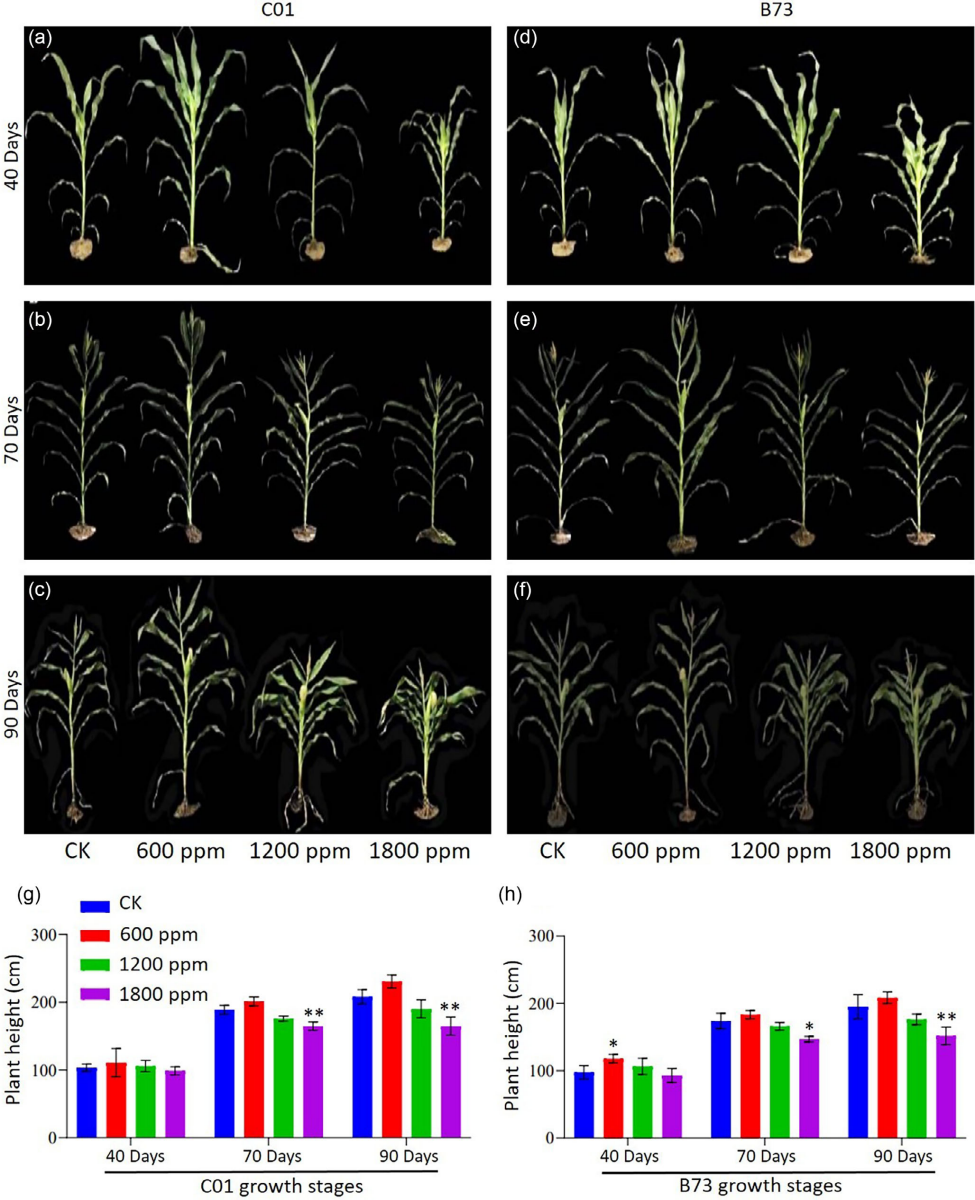
The effect of elevated CO_2_ on the growth of C01 and B73 maize genotypes. (a–c) Showing C01 at 40, 70 and 90 days after sowing (DAS). (d–f) Showing B73 at the same growth stages under ambient (CK), 600 ppm, 1200 ppm and 1800 ppm CO_2_. (g, h) The corresponding plant height measurements of C01 and B73 at 40, 70 and 90 DAS. Asterisks indicate significant differences compared to the control: *P < 0.05 and **P < 0.01.

The maize inbred lines C01 and B73 exhibited significant morphological variation in response to elevated CO_2_ (Fig. 2). At 40 DAS, both lines showed increased plant height under 600 ppm and 1200 ppm compared to ambient CO_2_, reaching 110.83 cm (C01) and 117.83 cm (B73) at 600 ppm, and 105.83 cm (C01) and 106.44 cm (B73) at 1200 ppm. By 70 DAS, maximum heights were recorded at 600 ppm (201.38 cm for C01; 183.27 cm for B73), exceeding the control values of 194.88 and 171.92 cm, respectively. At 90 DAS, plants at 600 ppm remained tallest, whereas those at 1200 ppm and 1800 ppm showed reduced height. Notably, at 1800 ppm, plant height declined significantly at 70 and 90 DAS, suggesting a threshold beyond which elevated CO_2_ suppresses growth.

Leaf area development exhibited genotype‐specific responses to elevated CO_2_ concentrations across different growth stages (40, 70 and 90 DAS) (Tables 2 and 3). In the C01 genotype, leaf area at 40 DAS was highest at 600 ppm CO_2_ but decreased at 1200 and 1800 ppm. By 70 DAS, the leaf area increased under 1200 ppm CO_2_ but was reduced at 600 and 1800 ppm. At 90 DAS, leaf area declined further compared to 70 DAS, with 600 ppm CO_2_‐treated plants showing the highest leaf area increase at this stage. Leaf, stem, and total dry biomass also followed a consistent trend across growth stages. The highest biomass values were observed at 600 ppm CO_2_, while reductions occurred at 1200 ppm and 1800 ppm CO_2_ (Table 2). At 40 DAS, the leaf area was greatest at 600 ppm CO_2_ for both C01 (693.04 cm^2^) and B73 (656.90 cm^2^). Leaf area at 600 ppm CO_2_ exceeded that at 1200 ppm and 1800 ppm CO_2_ (652.12 and 526.44 cm^2^ for C01; 610.80 and 513.51 cm^2^ for B73). By 90 DAS, leaf area growth was limited as a result of photosynthetic acclimation and leaf senescence. However, by 90 DAS, leaf area growth was limited due to photosynthetic acclimation and leaf senescence (Tables 2 and 3). Both genotypes, C01 and B73, followed similar trends. Plants treated with 600 ppm CO_2_ at 40, 70 and 90 DAS consistently exhibited the highest leaf, stem and total dry biomass compared to those treated with 1200 and 1800 ppm CO_2_. Interestingly, at 70 DAS, 1200 ppm CO_2_‐treated plants displayed the highest leaf area, whereas, at 90 DAS, leaf area was reduced for 600 and 1800 ppm CO_2_ treatments relative to 1200 ppm.

**Table 2:** Effects of elevated CO_2_ on dry biomass and leaf area accumulation in maize plants at different growth stages

Time intervals	Plant organ	600 ppm	1200 ppm	1800 ppm
40 DAS	Leaf (g)	12.75 (0.51) a	11.5 (0.52) b	9.5 (0.52) c
	Stem (g)	22.5 (0.84) a	18.0 (0.66) b	17.25 (1.1) b
	Total (g)	35.25 (1.36) a	29.5 (1.19) b	22.75 (1.04) d
	Leaf area (cm)	693.04 (1.7) a	652.12 (0.87) a	526.44 (0.21) c
70 DAS	Leaf (g)	39.25 (1.7) a	33.0 (0.47) b	28.25 (0.61) b
	Stem (g)	79.75 (1.54) a	65.75 (2.52) b	62 (5.26) b
	Total (g)	119 (3.1) a	98.75 (2.99) b	96 (1.8) b
	Leaf area (cm)	1325.8 (2.1) a	1335.28 (1.1) a	1244.6 (2.38) ab
90 DAS	Leaf (g)	32.75 (1.52) a	28.25 (0.61) b	26.25 (1.12) b
	Stem (g)	107.13 (1.98) b	98.35 (2.63) a	93.225 (1.44) b
	Total (g)	130.6 (3.5) b	135.38 (3.05) a	122.4 (2.62) bc
	Leaf area (cm)	1248 (3.1) a	1201.6 (2.1) ab	1122.74 (2.27) bc

Note: Values in parentheses are the standard error. Different lowercase letters within the same row indicate statistically significant differences (*P* < 0.05).

**Table 3:** Effect of elevated CO_2_ on dry biomass and leaf area in B73 maize plants

Growth stages	Plant organ	600 ppm	1200 ppm	1800 ppm
40 DAS	Leaf (g)	13.5 (0.52) a	10.5 (0.52) bc	8 (0.33) d
	Stem (g)	16 (0.66) bc	15.5 (0.70) bc	12 (1.05) d
	Total (g)	29.5 (1.17) b	26 (0.74) c	20 (1.3) e
	Leaf area (cm)	656.90 (2.4) a	610.80 (1.19) ab	513.51 (0.92) c
70 DAS	Leaf (g)	24.25 (0.51) c	25.5 (1.71) c	25 (1.12) e
	Stem (g)	48.75 (0.51) b	42.25 (1.98) d	37 (1.22) c
	Total (g)	73 (0.83) cd	67.75 (2.71) dc	62 (2.31) de
	Leaf area (cm)	1181.4 (1.6) b	1264.62 (1.48) ab	992.94 (1.45) c
90 DAS	Leaf(g)	27.7 (2.02) abc	29.25 (2.44) c	26.37 (1.23) abc
	Stem (g)	70.75 (2.45) d	99.5 (2.89) b	57.62 (23.0) c
	Total (g)	98.47 (4.7) d	128.925 (5.34) b	83.97 (4.27) e
	Leaf area (cm)	1093.18 (0.11) bcd	1074.74 (2.48) cd	850.44 (2.18) de

Note: Values in parentheses are the standard error. Different lowercase letters within the same row indicate statistically significant differences (*P* < 0.05).

### Effect of elevated CO_2_ on starch, soluble sugar, and TNC accumulation in maize

**Figure 3:**
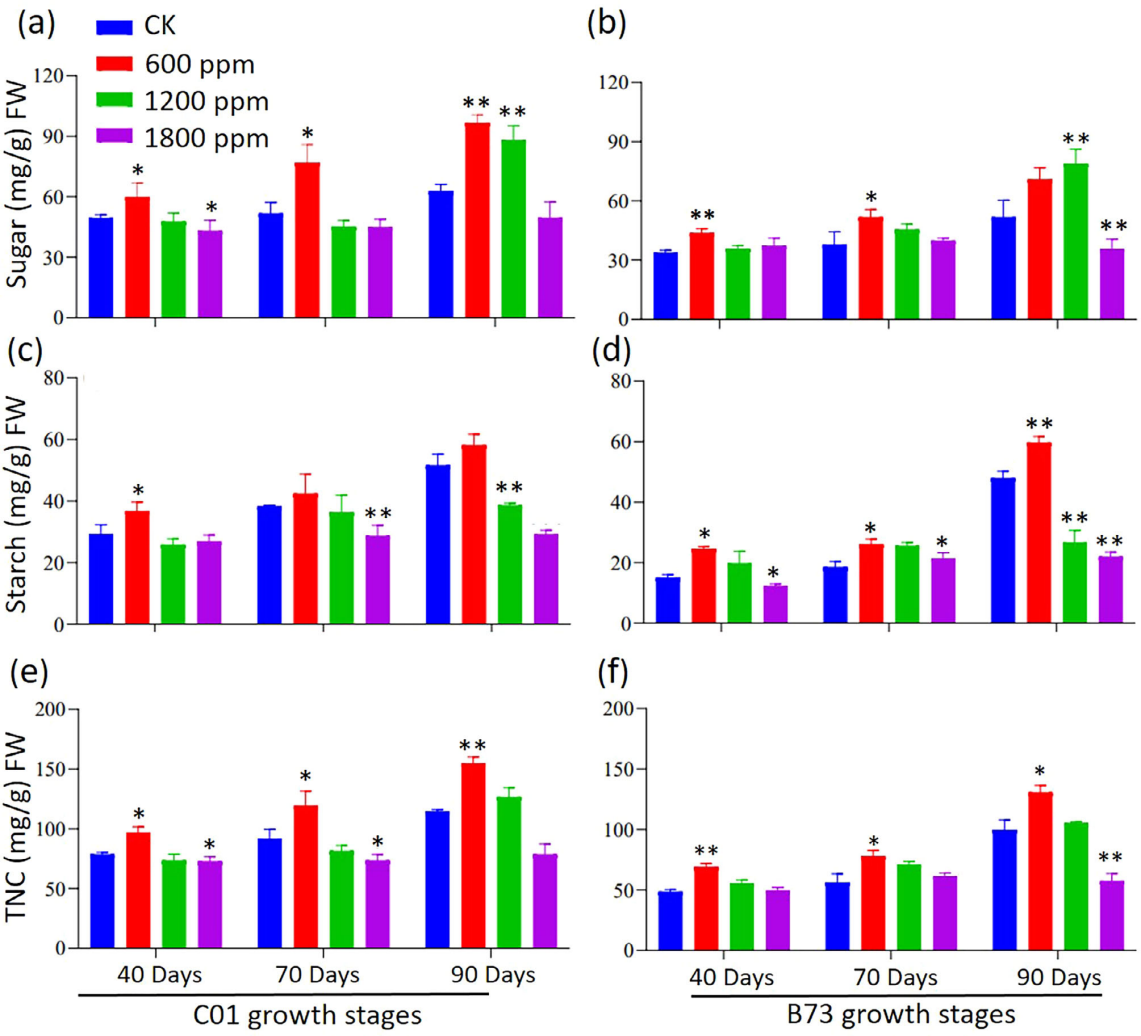
Effect of elevated CO_2_ on starch, soluble sugar, and total non‐structural carbohydrates in C01 and B73 maize leaves at different growth stages. (a,c,e) Showing the results for C01 maize. (b,d,f) Showing the data for B73 maize. Measurements were taken at different growth stages. Asterisks indicate significant differences compared to the control: *P < 0.05 and **P < 0.01.

The starch, soluble sugars and TNC were analyzed in the C01 and B73 genotypes grown under ambient and elevated CO_2_ conditions at 40, 70 and 90 DAS (Fig. 3). Across most stages, C01 exhibited higher sugar, starch and TNC levels than B73. In C01, sugar content significantly increased under 600 ppm CO_2_ at all stages, and 1200 ppm CO_2_ also enhanced sugar at 90 DAS (Fig. 3(a),(b)). In B73, sugar content increased significantly under 600 ppm CO_2_ at 40 and 70 DAS and under 1200 ppm CO_2_ at 90 DAS, whereas 1800 ppm CO_2_ significantly reduced sugar at 90 DAS (Fig. 3(b)). Starch content in C01 increased under 600 ppm CO_2_ at 40 DAS but decreased under 1800 ppm CO_2_ at 70 DAS and 1200 ppm CO_2_ at 90 DAS, whereas, in B73, starch increased under 600 ppm CO_2_ at all stages, whereas 1800 ppm CO_2_ caused reductions at all time points (Fig. 3(c),(d)). TNC levels were significantly enhanced under 600 ppm CO_2_ at all stages in both genotypes, whereas 1800 ppm CO_2_ inhibited TNC accumulation (Fig. 3(e),(f)). These results indicate that elevated CO_2_ differentially affects carbohydrate metabolism, with 600 ppm CO_2_ consistently promoting sugar, starch and TNC accumulation, whereas 1800 ppm CO_2_ exerts inhibitory effects, particularly at 90 DAS.

### Impact of elevated CO_2_ on photosynthetic pigments in maize

Chlorophyll and carotenoid contents in maize were significantly affected by elevated CO_2_, with notable differences between the C01 and B73 varieties (Tables 4 and 5). In C01, chlorophyll *a*, chlorophyll *b* and carotenoids decreased progressively with increasing CO_2_ concentrations (600, 1200 and 1800 ppm) across all three harvesting stages (Table 4). The most pronounced reductions were observed at 1800 ppm, indicating heightened sensitivity of C01 to elevated CO_2_ during reproductive development. Similarly, in the B73 variety, chlorophyll *a* chlorophyll *b* and carotenoids showed a consistent reduction with increasing CO_2_ levels across all three harvesting stages (Table 5). Among the tested concentrations, 1800 ppm caused the greatest reduction in all photosynthetic pigments. Comparative analysis of the two varieties revealed that B73 maintained higher chlorophyll and carotenoid levels than C01 under both ambient and elevated CO_2_ conditions, suggesting a greater resilience of B73 to CO_2_‐induced stress.

**Table 4:** Effect of elevated CO_2_ on chlorophyll and carotenoid in C01 maize plants

Growth stages	CO_2_ conccentration	Chlorophyll *a*	Chlorophyll *b*	Carotenoid
40 DAS	CK	12.86 (0.67) a	2.66 (0.08) a	4.59 (0.08) a
	600 ppm	12.28 (0.94) ab	1.78 (0.78) a	3.69 (0.78) abc
	1200 ppm	11.29 (0.39) abc	1.36 (0.34) a	2.83 (0.34) bed
	1800 ppm	10.10 (0.42) bc	0.85 (0.15) a	2.56 (0.15) c
70 DAS	CK	13.75 (0.26) d	2.98 (0.52) a	5.49 (0.52) a
	600 ppm	11.03 (0.19) bc	1.97 (0.33) ab	4.08 (0.33) b
	1200 ppm	9.96 (0.15) cd	1.68 (0.2) ab	3.75 (0.2) bc
	1800 ppm	9.58 (0.55) d	1.22 (0.21) ab	2.86 (0.21) bc
90 DAS	CK	11 (1.42) a	2.58 (0.45) a	3.66 (0.45) a
	600 ppm	9.3 (0.37) a	1.5 (0.39) a	2.15 (0.39) bcd
	1200 ppm	6.42 (0.11) b	0.96 (0.57) a	1.77 (0.57) cd
	1800 ppm	4.23 (0.39) c	0.58 (0.63) a	1.76 (0.63) cd

Note: Values in parentheses are the standard error. Different lowercase letters within the same row indicate statistically significant differences (*P* < 0.05).

**Table 5:** Effect of elevated CO_2_ on chlorophyll and carotenoid in B73 maize plants

Growth stages	CO_2_ concentration	Chlorophyll *a*	Chlorophyll *b*	Carotenoid
40 DAS	CK	13.10 (1.38) a	1.96 (0.99) a	4.49 (0.49) a
	600 ppm	11.47 (0.47) ab	1.49 (0.69) b	3.85 (0.39) ab
	1200 ppm	10.39 (1.13) bc	0.97 (1.44) a	3.97 (0.57) ab
	1800 ppm	9.28 (0.22) c	0.43 (0.26) b	2.48 (0.30) d
70 DAS	CK	13.63 (0.74) a	2.21 (0.43) ab	4.04 (0.43) a
	600 ppm	11.03 (0.19) bc	1.84 (0.80) ab	3.34 (0.67) bc
	1200 ppm	10.42 (0.42) bc	1.62 (0.12) b	3.51 (0.26) bc
	1800 ppm	9.59 (0.68) d	0.84 (0.80) b	2.71 (0.65) c
90 DAS	CK	9.52 (0.82) a	1.86 (1.08) a	3.40 (0.10) a
	600 ppm	6.07 (1.47) bc	1.07 (1.54) a	2.98 (0.41) ab
	1200 ppm	5.05 (0.08) bc	0.97 (0.09) a	1.83 (0.09) cd
	1800 ppm	4.08 (0.52)c	0.40 (0.40) a	1.34 (0.04) d

Note: Values in parentheses are the standard error. Different lowwercase letters within the same row indicate statistically significant differences (*P* < 0.05).

### Effects of elevated CO_2_ on folate content and derivatives in maize: differential responses in C01 and B73

**Figure 4:**
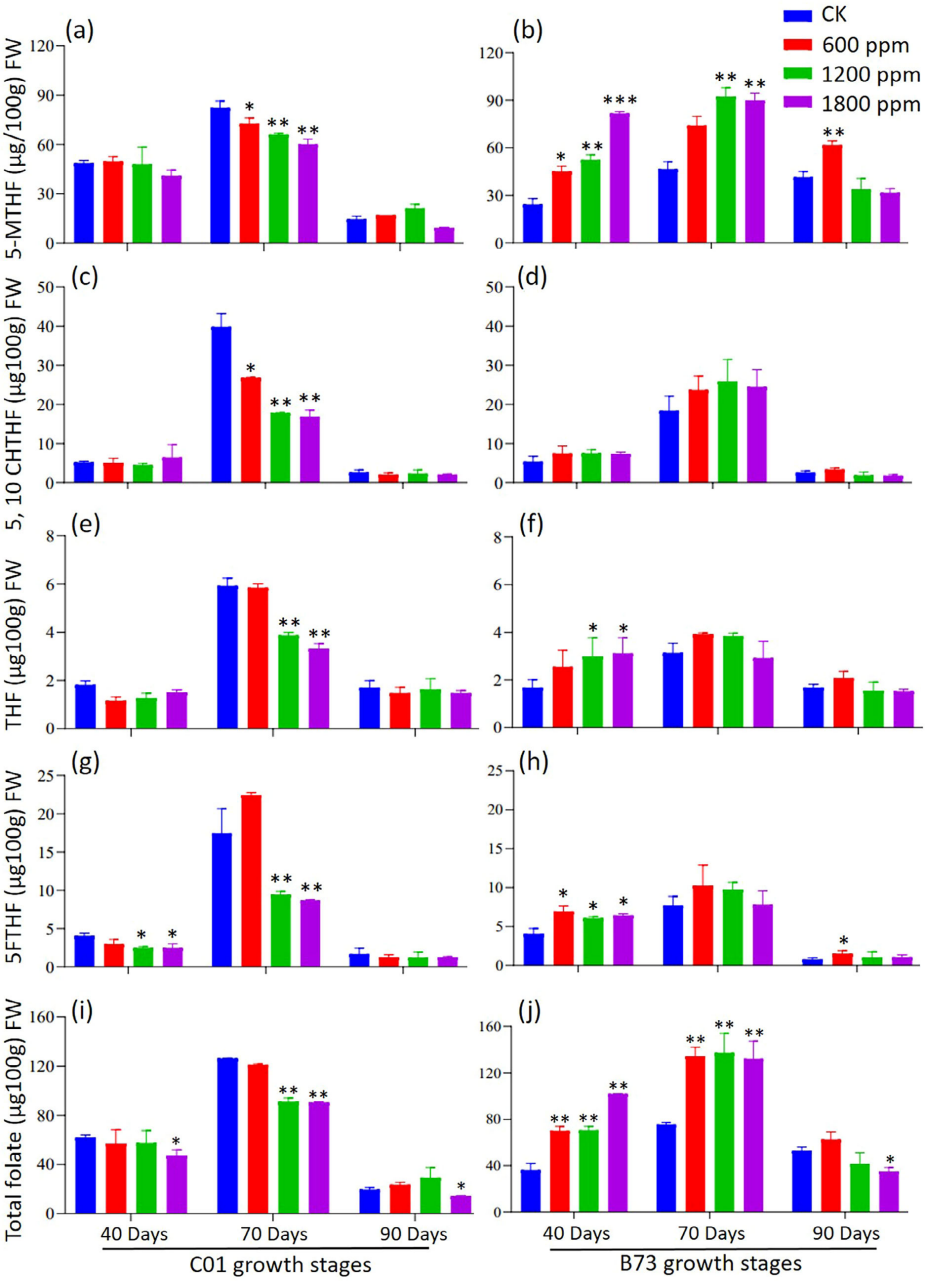
Effect of elevated CO_2_ on folate derivatives in C01 and B73 maize at various growth stages. (a,c,e,g,i) Showing data for C01 maize. (b,d,f,h,j) Showing data for B73 maize. Asterisks indicate significant differences compared to the control: *P < 0.05, **P < 0.01 and ***P < 0.001.

The total folate content and its derivatives in C01 and B73 varied across developmental stages and CO_2_ treatments (Fig. 4). Levels of 5‐methyltetrahydrofolate (5MTHF) significantly increased at 70 DAS but declined at 90 DAS (Fig. 4(a)). Under elevated CO_2_ conditions, 5MTHF levels were higher in B73 compared to C01. At 40 DAS, 5MTHF concentrations in B73 increased by 85%, 16% and 37% at 600, 1200 and 1800 ppm CO_2_, respectively (Fig. 4((b))). At 70 DAS, significant increases occurred at 1200 and 1800 ppm (52% over control), whereas, at 90 DAS, significant elevation was observed only at 600 ppm. 5,10‐Methenyltetrahydrofolate (5,10CHTHF) peaked at 70 DAS in both genotypes (Fig. 4(c),(d)). In C01, levels declined with rising CO_2_, whereas, in B73, levels increased at all concentrations but were not statistically significant. Similarly, tetrahydrofolate (THF), 5‐formyltetrahydrofolate (5FTHF) and total folate were highest at 70 DAS. In C01, THF and 5FTHF decreased under 1200 and 1800 ppm CO_2_ at 70 DAS (Fig. 4(e),(g)), whereas B73 showed significant increases at all elevated CO_2_ levels (Fig. 4(f),(h)). Total folate exhibited opposing trends: C01 decreased under elevated CO_2_, whereas B73 increased (Fig. 4(i),(j)). Folate content rose during growth, peaking at 70 DAS, then declined at maturity. At 90 DAS, B73 had higher folate than C01. Elevated CO_2_ significantly enhanced folate in B73 at 40 and 90 DAS, with increases of 72%, 95% and 153% at 600, 1200 and 1800 ppm at 40 DAS, respectively. No significant changes occurred in C01.

### Effects of elevated CO_2_ on lignin content in maize

**Figure 5:**
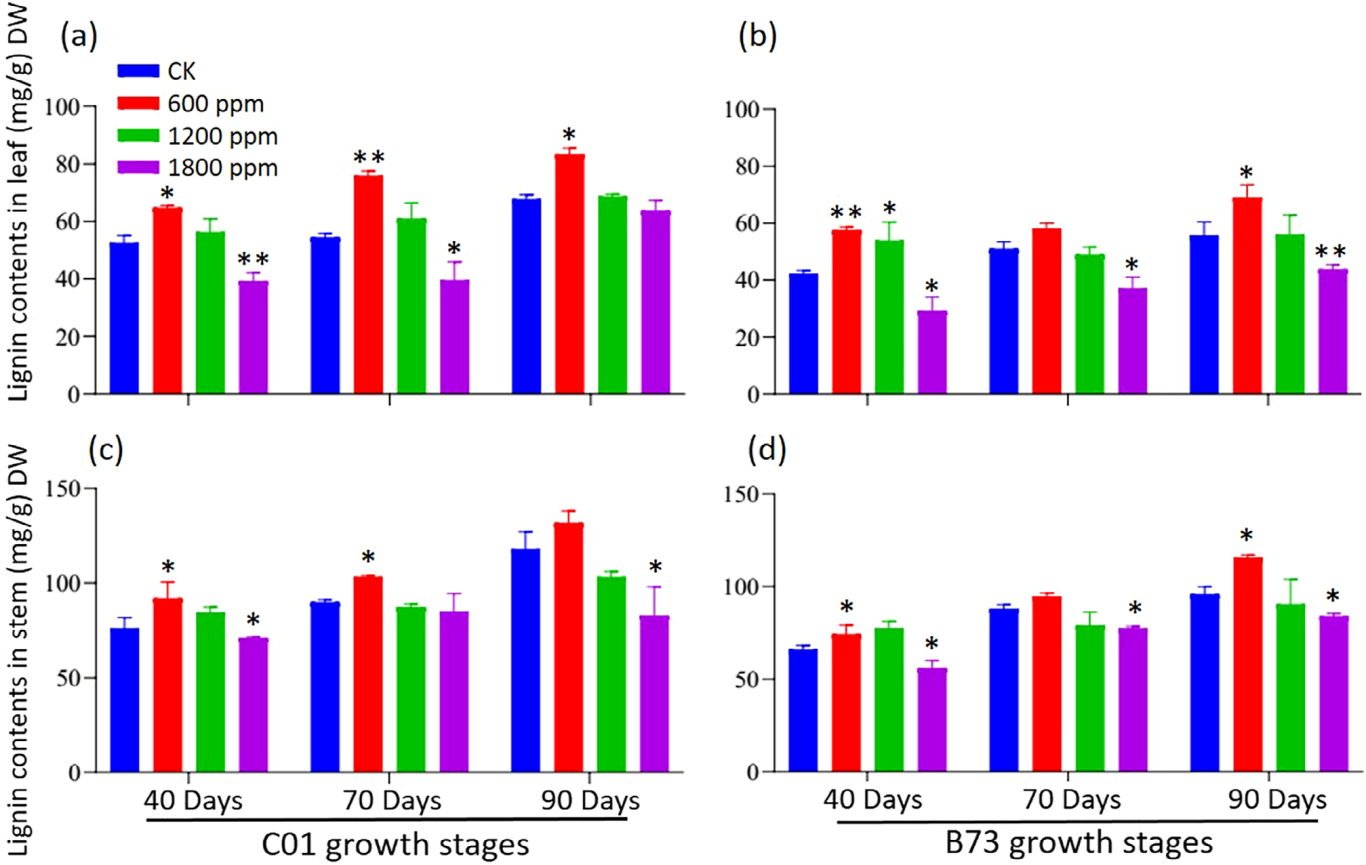
Effect of elevated CO_2_ on lignin content in C01 and B73 maize leaf and stem tissues at 40, 70 and 90 DAS. (a,c) Showing data for C01 maize leaf and stem respectively, whereas panels (b,d) present data for B73 maize leaf and stem respectively. Asterisks indicate significant differences compared to the control: *P < 0.05 and **P < 0.01.

This study investigated lignin content in C01 and B73 maize under varying CO_2_ concentrations at 40, 70 and 90 DAS (Fig. 5). Patterns of lignin accumulation varied with CO_2_ levels and tissue types. Under ambient CO_2_, lignin content steadily increased over time. By contrast, elevated CO_2_ significantly reduced lignin levels in both leaves and stems. At 600 ppm CO_2_, lignin content increased at 40, 70 and 90 DAS in both the leaves and stems of C01 and B73 plants compared to their respective controls. However, at 1800 ppm CO_2_, lignin content was significantly reduced at all three stages in both tissues of both maize varieties. Across all growth stages, stems consistently exhibited higher lignin content than leaves. Elevated CO_2_ showed an inverse relationship with lignin levels, with higher CO_2_ concentrations causing greater reductions in lignin accumulation. The impact was more pronounced in leaves than stems, likely reflecting tissue‐specific responses to elevated CO_2_. These findings suggest that elevated CO_2_ suppresses lignin biosynthesis in maize, with stems maintaining relatively higher lignin levels than leaves. The results underscore the potential implications of rising atmospheric CO_2_ on plant structural integrity, stress resilience and biomass composition.

### Dynamic changes in lignin monomers and S/G ratio under elevated CO_2_

Elevated CO_2_ had a dynamic impact on lignin composition in the leaves and stems of C01 and B73, as shown in Tables 6, 7, 8. At 40 DAS, total lignin monomers were generally reduced under elevated CO_2_, although minor increases were observed in guaiacyl (G) lignin in C01 leaves and hydroxyphenyl (H) lignin in B73 leaves (Table 6). The syringyl‐to‐guaiacyl (S/G) ratio significantly decreased in C01 leaves but increased in B73 stems relative to control plants. At 70 DAS, elevated CO_2_ reduced H lignin and the S/G ratio in C01 leaves; G lignin, S lignin and the S/G ratio in B73 leaves; H, G and S lignin in C01 stems; and H lignin in B73 stems, compared to control plants (Table 7). Conversely, G and S lignin in C01 leaves, H lignin in B73 leaves and the S/G ratio in C01 stems increased under elevated CO_2_. In B73 stems, G and S lignin and the S/G ratio increased at 600 ppm CO_2_ compared to control plants but declined at 1200 and 1800 ppm CO_2_ relative to the 600 ppm treatment. By 90 DAS, elevated CO_2_ generally reduced H, G and S lignin in C01 stems and H lignin in B73 stems, whereas G and S lignin and the S/G ratio increased in C01 leaves and stems and in B73 stems. However, H lignin in B73 stems showed a slight increase at 1200 ppm CO_2_ (Table 8). By contrast, G and S lignin and the S/G ratio in C01 leaves, the S/G ratio in C01 stems and the S/G ratio in B73 stems increased under elevated CO_2_. Notably, S and the S/G ratio in C01 leaves showed slight reductions at higher CO_2_ levels. Additionally, in B73 leaves, H, G and S lignin increased at 600 ppm CO_2_ compared to control plants but declined at higher CO_2_ concentrations. Similarly, G and S lignin in B73 stems followed a comparable trend. These results indicate that the effects of elevated CO_2_ on lignin composition are both tissue‐ and growth‐stage‐specific, with varying responses depending on the CO_2_ concentration and genetic background of the plants.

**Table 6:** Effect of elevated CO_2_ on lignin biosynthesis in C01 and B73 line in leaf and stem at 40 DAS

Plant organ	C01/B73	Lignin monomers (μmol mg^−1^)	CO_2_ treatment			
			CK	600 ppm	1200 ppm	1800 ppm
Leaf	C01	H	28.07 (0.19) c	65.55 (0.45) b	28 (1.02) c	5.35 (0.21) d
		G	832 (16.4) b	1042.2 (4.5) a	1145.7 (13.12) a	862.37 (5.58) b
		S	81.64 (1.50) f	224.6 (1.11) a	184.57 (2.7) b	31.67 (6.30) g
		S/G	0.9 (0.02) d	0.21 (0.015) a	0.15 (0.01) c	0.03 (0.007) c
	B73	H	25.78 (1.12) c	68.610 (0.9) a	27.4 (0.18) c	26.42 (0.52) c
		G	536.04 (16) d	551.91 (9.2) d	657.83 (3.10) c	493.89 (12.2) d
		S	120 (6.9) de	111.68 (3.2) d	106.55 (1.75) c	94.47 (2.79) c
		S/G	0.19 (0.08) b	0.2 (0.002) ab	0.2 (0.002) ab	0.19 (0.007) b
Stem	C01	H	10.8 (0.19) b	6.7 (0.002) d	1.88 (0.05) c	1.16 (0.04) c
		G	353.28 (2.7) c	261.6 (1.70) c	349.64 (18) cd	270.03 (7.95) c
		S	489.84 (7.1) c	459.2 (17.8) c	273.91 (16) f	357.85 (11.5) d
		S/G	1.56 (0.1) ab	1.75 (0.06) a	0.78 (0.05) de	1.32 0.003 c
	B73	H	8.34 (0.17) c	10.65 (0.13) a	20.42 (0.16) a	10.24 (0.05) b
		G	900.8 (10.3) a	913.65 (6.2) a	828.88 (3.96) d	799.6 (9.02) b
		S	540.9 (1.2) b	1315.5 (10) a	860.26 (6.42) c	525.76 (10) b
		S/G	0.6 (0.005) e	1.43 (0.01) bc	0.95 (0.008) d	0.65 (0.005) c

Note: Values in parentheses are the standard error. Different lowercase letters within the same row indicate statistically significant differences (*P* < 0.05). H, *p*‐hydroxyphenyl; G, guaiacyl; S, syringly; S/G, syringly/guaiacyl.

**Table 7:** Effect of elevated CO_2_ on lignin biosynthesis in C01 and B73 line in leaf and stem at 70 DAS.

Plant organ	C01/B73	Lignin monomers (μmol mg^−1^)	CO_2_ treatment			
			CK	600 ppm	1200 ppm	1800 ppm
Leaf	C01	H	17.17 (0.19) c	7.7 (0.31) e	6.02 (0.07) e	2.81 (0.09) f
		G	517.8 (18.3) c	706.5 (5.02) c	1070 (15.84) b	843.02 (19.6) d
		S	123.2 (11.7) e	187.7 (14.6) d	235.74 (4.85) c	123.33 (18) e
		S/G	0.33 (0.01) d	0.23 (0.04) ab	0.22 (0.006) d	0.14 (0.02) c
	B73	H	12.6 (0.88) d	39.21 (0.58) a	40.73 (1.02) a	27.743 (0.12) b
		G	1120.8 (1.3) a	955.3 (0.52) c	821.58 (9.6) d	816.21 (3) d
		S	354.72 (5.7) a	305.09 (3.5) b	292.34 (19) b	254.72 (3.08) c
		S/G	0.37 (0.005) a	0.37 (0.01) c	0.26 (0.02) cd	0.31 (0.01) bc
Stem	C01	H	8.68 (0.57) d	4.58 (0.11) ef	3.68 (0.16) f	4.68 (0.11) ef
		G	1518.6 (9.9) a	1281.8 (16) b	807.22 (3.16) f	732.55 (10.1) g
		S	2356.6 (20) a	1625.2 (12) b	1503 (0.8) c	1511 (16.4) c
		S/G	1.55 (0.01) c	1.3 (0.04) d	1.86 (0.007) b	2.06 (0.05) a
	B73	H	24.35 (0.73) b	20.98 (1.02) a	13 (1.14) c	6.193 (0.11) e
		G	902.74 (5.6) c	1187.4 (1.2) b	1081.2 (17) d	789.15 (9.41) f
		S	820.45 (16.3) f	1327.8 (7.5) d	1140.02 (20) e	832.89 (3.90) f
		S/G	0.9 (0.02) f	1.11 (0.04) c	1.05 (0.03) c	1.02 (0.02) e

Note: Values in parentheses are the standard error. Different lowercase letters within the same row indicate statistically significant differences (*P* < 0.05). H, *p*‐hydroxyphenyl; G, guaiacyl; S, syringly; S/G, syringly/guaiacyl.

**Table 8:** Effect of elevated CO_2_ on lignin biosynthesis in C01 and B73 line in leaf and stem at 90 DAS.

Plant organ	C01/B73	Lignin monomers (μmol mg^−1^)	CO_2_ treatment			
			CK	600 ppm	1200 ppm	1800 ppm
Leaf	C01	H	10.24 (1) d	2.36 (0.08) e	1.72 (0.07) e	2.2 (0.04) e
		G	419.2 (0.39) f	430.83 (15) f	575.9 (3.33) e	715.29 (7.65) c
		S	71.62 (8.32) f	103.7 (12.8) f	80.29 (6.33) f	311.76 (4.95) d
		S/G	0.17 (0.19) c	0.23 (0.02) c	0.16 (0.01) c	0.43 (0.04) b
	B73	H	26.5 (0.36) a	56.5 (0.32) b	35 (0.42) c	27.75 (0.40) c
		G	404.7 (13.8) f	1261.9 (9.1) a	991.5 (11.9) b	658.18 (13.9) d
		S	263.6 (17.3) e	540.74 (4.9) a	454.4 (15.3) b	413.15 (2.39) c
		S/G	0.63 (0.06) a	0.42 (0.01) b	0.45 (0.01) b	0.62 (0.01) a
Stem	C01	H	12.17 (0.52) a	8.84 (0.27) f	5.49 (0.05) f	3.14 (0.02) h
		G	1171.5 (2.3) a	1050.6 (19) b	943.81 (9.7) d	740.85 (2.60) f
		S	2043.8 (8.9) a	1946.2 (6.2) b	1644.4 (19) c	1420.9 (17.5) d
		S/G	1.74 (0.07) b	1.85 (0.036) a	1.74 (0.038) b	1.81 (0.02) a
	B73	H	19.36 (0.29) b	14.57 (0.06) c	34.6 (11.34) f	8.76 (0.05) e
		G	927.56 (6.7) d	1013.1 (1.4) c	879.65 (6.6) e	697.25 (8.5) g
		S	1139.4 (19) f	1300.1 (6.3) e	1182.5 (4.6) c	1010 (15.29) g
		S/G	1.22 (0.15) d	1.28 (0.06) cd	1.34 (0.05) b	1.69 (0.05) b

Note: Values in parentheses are the standard error. Different lowercase letters within the same row indicate statistically significant differences (*P* < 0.05). H, *p*‐hydroxyphenyl; G, guaiacyl; S, syringly; S/G, syringly/guaiacyl.

### Histochemical staining of maize leaf under ambient and elevated CO_2_ treatments

**Figure 6:**
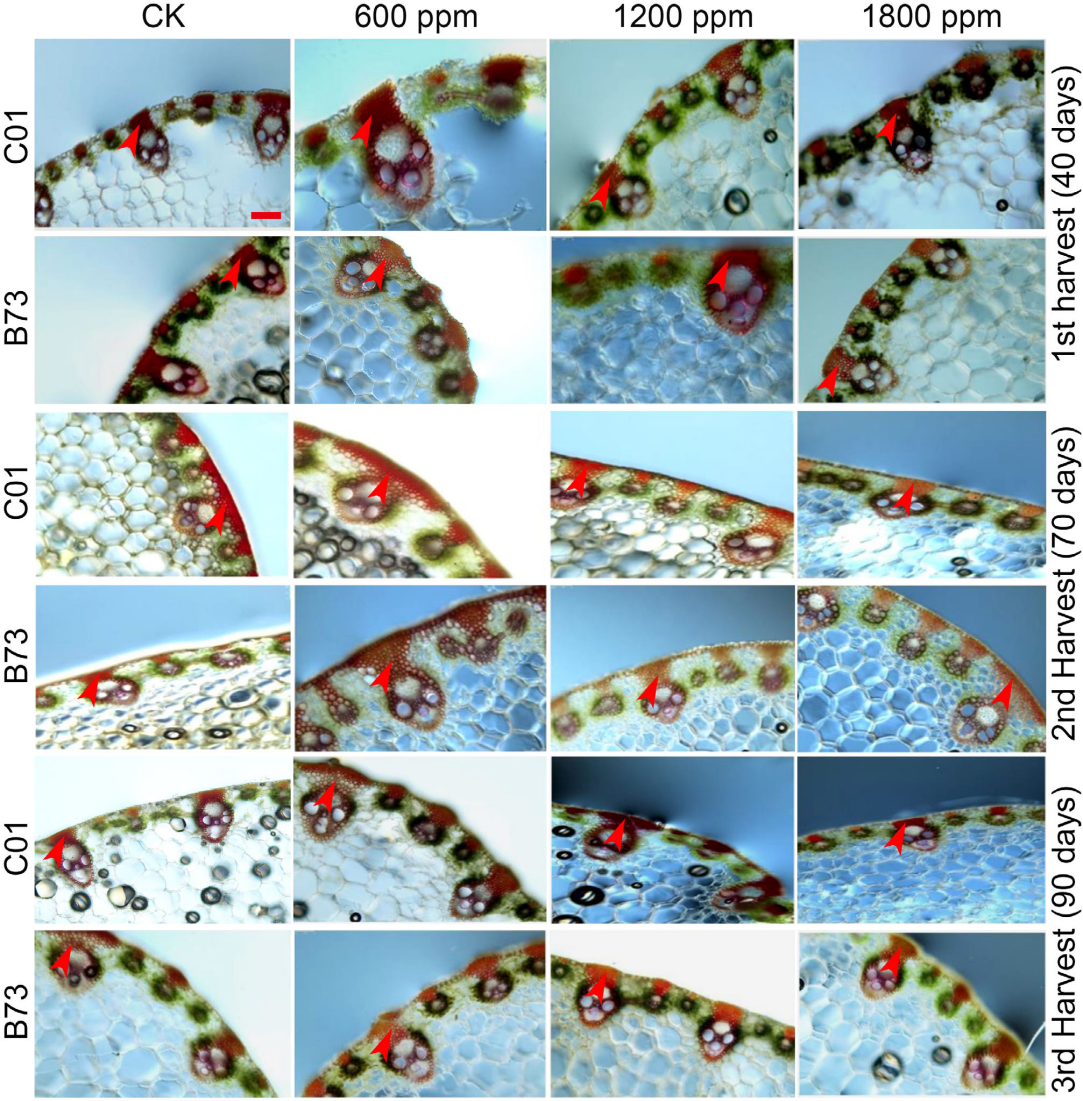
Histochemical staining of lignin in leaf sections from C01 and B73 maize. Midrib sections from the first harvest (40 DAS), second harvest (70 DAS) and third harvest (90 DAS) were collected from both C01 and B73 maize plants and stained with phloroglucinol to visualize lignin content. Arrows indicate lignin deposition.

Sections of the midrib from both C01 and B73 maize plants were stained with acid phloroglucinol to visualize lignin deposition (Fig. 6). In both genotypes, the midrib, epidermis, and tissues surrounding the vascular bundles exhibited red staining at lower levels of elevated CO_2_, indicating active lignification. However, at higher CO_2_ concentrations (1200 ppm and 1800 ppm), the staining intensity was noticeably diminished in both C01 and B73, reflecting a significant reduction in lignification. Although no discernible anatomical changes were observed in the midrib structures of either genotype, marked differences in staining intensity were evident. C01 plants consistently displayed stronger phloroglucinol staining, suggesting that lignin content was inherently higher in C01. Additionally, staining intensity varied with plant age, as observed at 40, 70 and 90 DAS, indicating that lignification was influenced by both developmental stage and CO_2_ concentration. These findings suggest genotype‐specific responses to elevated CO_2_ in lignin biosynthesis and deposition.

### Expression patterns of lignin biosynthesis genes in maize under elevated CO_2_ conditions

**Figure 7:**
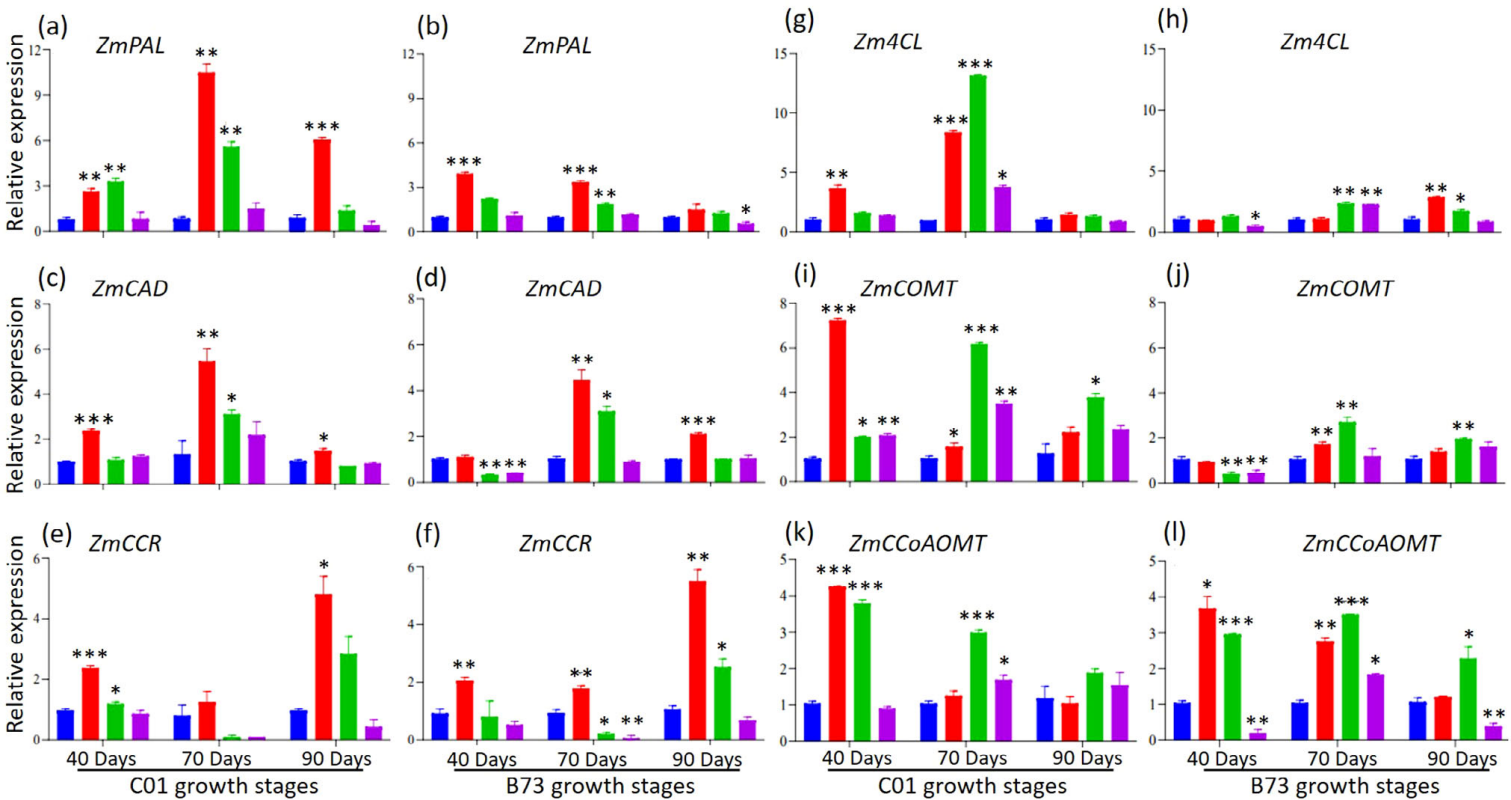
Effect of elevated CO_2_ on lignin biosynthesis genes in C01 and B73 maize at various growth stages. (a,c,e,g,i,k) Showing data for C01 maize. (b,d,f,h,j,l) Showing data for B73 maize. Asterisks indicate significant differences compared to the control: *P < 0.05, **P < 0.01 and ***P < 0.001.

Elevated CO_2_ altered lignin composition and gene expression. In C01 plants, phenylalanine ammonia‐lyase (PAL) was strongly upregulated, with the highest expression at 600 ppm and the lowest at 1800 ppm (Fig. 7(a)). In B73, *ZmPAL* increased at 40 and 70 DAS under 600 ppm but declined sharply at 90 DAS under 1800 ppm (Fig. 7(b)) and overall transcript levels were lower than in C01 (Fig. 7(a),(b)). *ZmCAD* and *ZmCCR* showed similar patterns in both genotypes, increasing under 600 ppm at all stages (Fig. 7(c)–(f)). *ZmCAD* peaked at 70 DAS and declined at 90 DAS, whereas *ZmCCR* was highest at 90 DAS. In B73, *ZmCAD* was reduced at 40 DAS under 1200 and 1800 ppm, and *ZmCCR* was suppressed at 70 DAS under the same conditions. *Zm4CL* was strongly upregulated in C01 at 40 and 70 DAS, particularly under 600 ppm, and remained higher than in B73 across treatments (Fig. 7(g),(h)). In B73, expression increased mainly at 70 and 90 DAS under 1200 and 1800 ppm. *ZmCOMT* expression in C01 was highest at 40 DAS under 600 ppm, peaked at 70 DAS under 1200 ppm, and increased again at 90 DAS under 1200 ppm (Fig. 7(i)). In B73, expression was inhibited at 40 DAS under 1200 and 1800 ppm but increased at 70 DAS under 600 and 1200 ppm (Fig. 7(j)). Overall, *ZmCOMT* levels were consistently higher in C01. *ZmCCoAOMT* in C01 peaked at 40 DAS under 600 ppm and at 70 DAS under 1200 ppm, with expression remaining higher than in B73 (Fig. 7(k)). In B73, the pattern was similar at 40 DAS, but, at 70 DAS, the highest expression occurred under 1200 ppm. At 90 DAS, expression was strongly reduced under 1800 ppm (Fig. 7(l)).

## DISCUSSION

### Genotype‐specific effects of elevated CO_2_

The present study reveals the genotype‐specific effect of elevated CO_2_ on maize growth, lignin biosynthesis and folate metabolism. Moderate CO_2_ levels (600 ppm) enhanced growth and biomass, whereas higher levels (1200 and 1800 ppm) caused physiological imbalance, including reduced pigment synthesis and disrupted lignin accumulation. Genotypic differences were evident, with C01 showing higher lignin and sugar accumulation, and B73 exhibiting greater starch and folate levels. These findings highlight the complex interactions between elevated CO_2_ and plant metabolic processes, offering insight into genetic and physiological mechanisms that could inform future crop management under changing atmospheric conditions.

### Plant height and morphogenesis

Plant height is a critical determinant of crop morphogenesis and grain yield, directly influencing the yield index and quality. In this study, elevated CO_2_ significantly affected maize growth (Fig. 2), with the lowest plant height at 1800 ppm CO_2_ and the highest at 600 ppm CO_2_, indicating optimal growth under moderate CO_2_ enrichment. Previous studies report contrasting effects of elevated CO_2_ on maize height. For example, some found increased plant height,^34^ whereas others observed no significant change under similar conditions,^7^ reflecting complex CO_2_ response dynamics influenced by environmental conditions, genotypes, or methodologies.^5^ Moderate CO_2_ enrichment (600 ppm) also promoted the highest leaf area and total biomass, whereas excessive CO_2_ (1800 ppm) reduced growth, likely as a result of physiological constraints. Leaf area peaked at 40 DAS under 600 ppm CO_2_ and declined later because of senescence. Genotypic differences were evident, with B73 outperforming C01. These results align with previous studies showing that elevated CO_2_ enhances growth rates and leaf area,^35^, ^36^, ^37^, ^38^.^38^, ^39^ The improved growth observed at 600 ppm CO_2_ can be attributed to enhanced carbon assimilation and energy availability, which promote leaf expansion and biomass accumulation, as supported previously.^38^, ^40^ Conversely, the reduced growth at 1800 ppm CO_2_ likely reflects an imbalance in carbon metabolism, where excessive CO_2_ surpasses the photosynthetic machinery's capacity. This imbalance may lead to diminished carbohydrate breakdown and accelerated leaf senescence.^41^ These findings emphasize the importance of maintaining optimal CO_2_ levels. Although moderate enrichment (600 ppm) enhances maize growth and productivity, excessive CO_2_ concentrations impose physiological stress, underscoring the need for precise management of atmospheric CO_2_ levels to optimize crop performance.

### Carbohydrate metabolism and pigment changes

Soluble sugars and starches are the primary types of carbohydrates stored in plant vegetative tissues, such as stems and leaf sheaths.^42^ In the present study, C01 consistently exhibited higher sugar, starch and TNC levels than B73, reflecting genotypic differences in photosynthetic efficiency and carbohydrate metabolism. Sugar, starch and TNC levels increased significantly at 600 ppm CO_2_ in both genotypes, whereas higher CO_2_ concentrations reduced these carbohydrates suggesting a threshold beyond which photosynthetic downregulation limits carbohydrate production. At higher CO_2_ concentrations, plants often experience photosynthetic downregulation, which reduces enzymatic activity, decreases photosynthetic efficiency, and limits carbohydrate production. Similar observations have been reported in soybeans, where sugar, starch and TNC levels increased at 600 ppm CO_2_ but declined at CO_2_ concentrations ranging from 800 to 1600 ppm.^43^ Additionally, our study demonstrated that elevated CO_2_ reduced chlorophyll and carotenoid contents in both genotypes at all three time points (Tables 4 and 5). These results are consistent with previous studies showing that elevated CO_2_ inhibits chlorophyll accumulation in plants.^38^, ^44^, ^45^ The precise mechanism underlying chlorophyll reduction as a result of CO_2_ elevation remains unclear. However, it is hypothesized that the reduction may result from dilution effects caused by excessive carbohydrate accumulation. Wildman^46^ proposed that starch grain formation might cause chloroplast disorientation, leading to reduced light interception. Based on our findings, the decrease in chlorophyll content with increasing CO_2_ concentration may be attributed to chloroplast degeneration caused by starch accumulation, as suggested in other studies.^47^, ^48^ These results highlight the need for further investigation into the interplay between elevated CO_2_, carbohydrate metabolism and chloroplast structure to better understand the physiological responses of plants to changing atmospheric conditions.

### Folate metabolism and genotypic variability

Folate content and its derivatives in maize cultivars C01 and B73 varied significantly across developmental stages and CO_2_ levels, exhibiting distinct cultivar‐specific responses (Fig. 4). Folate levels peaked at 70 DAS before declining by 90 DAS. Elevated CO_2_ significantly enhanced 5‐methyltetrahydrofolate (5MTHF), tetrahydrofolate (THF), 5‐formyltetrahydrofolate (5FTHF) and total folate content in B73, particularly at 40 DAS and 90 DAS, with the highest increases observed at 1800 ppm CO_2_. By contrast, C01 displayed minimal or even negative responses to elevated CO_2_, especially at higher concentrations. Recent studies highlight the variable impact of elevated CO_2_ on folate and related metabolites. For example, a 30% reduction in folate accumulation was reported in rice under elevated CO_2_.^49^ It has been hypothesized that methylenetetrahydrofolate reductase (MTHFR) catalyzes the reduction of 5,10‐methylene‐THF to 5‐methyl‐THF. Reduced MTHFR expression in maize and tobacco lines has been linked to decreased cellular levels of 5‐methyl‐THF, whereas levels of more oxidized folate species either remain constant or increase.^50^ These observations suggest that elevated CO_2_ may perturb MTHFR enzyme activities, resulting in decreased 5‐methyl‐THF levels and fluctuations in other oxidized folates under varying CO_2_ treatments. Elevated CO_2_ has also been shown to influence mineral and vitamin content in rice,^49^, ^51^ and CO_2_‐induced folate variation may relate to declines in nitrogen content^6^ or photosynthetic depression, which reduces NADPH availability necessary for folate biosynthesis.^52^ Overall, the results emphasize the complex interplay between CO_2_ levels, folate metabolism and photosynthesis. Understanding these interactions can inform strategies to enhance folate biosynthesis in crops, thereby improving nutritional outcomes under future atmospheric CO_2_ scenarios.

### Lignin biosynthesis and structural integrity

The ‘source‐sink balance hypothesis’ posits that elevated CO_2_ or nutrient stress increases carbon availability, resulting in the accumulation of carbon‐based secondary or structural compounds in source leaves.^53^ Although the levels of secondary compounds generally rise under elevated CO_2_, their chemical composition often varies depending on the plant species or genotype.^54^ In the present study, elevated CO_2_ significantly reduced lignin content in both leaves and stems of maize varieties C01 and B73, with the extent of reduction increasing at higher CO_2_ levels (Fig. 5). Notably, lignin levels were consistently higher in stems than in leaves; however, the reduction caused by elevated CO_2_ was more pronounced in leaves. This suggests that elevated CO_2_ has the potential to suppress lignin biosynthesis, which may influence plant structural integrity and resilience to biotic and abiotic stresses. Interestingly, genotypic variation did not have a significant effect on lignin accumulation. The response to different CO_2_ levels was threshold‐dependent, with lignin accumulation observed at 600 ppm CO_2_ but a reduction noted at 1200 ppm and 1800 ppm CO_2_. These findings contrast with sorghum, where elevated CO_2_ increased lignin,^55^ and align with studies showing no effect on lignin or cellulose in soybean, shrubs or scrub oak.^56^ In maize, elevated CO_2_ levels appear to direct carbon to lignin biosynthesis up to 600 ppm, whereas higher CO_2_ levels might lead to a reallocation of resources to other metabolic pathways. Additionally, photoperiod could influence lignin biosynthesis under elevated CO_2_. Short days are characterized by increased carbon allocation to starch, which is synthesized rapidly during the day and slowly degraded at night. Conversely, long days favor sucrose production, resulting in slower starch synthesis and faster degradation, potentially altering carbon allocation to lignin biosynthesis^56^

### Lignin monomers and gene expression

Our findings were further supported by quantifying lignin monomers and evaluating the expression of genes involved in lignin biosynthesis. Elevated CO_2_ affected lignin composition in a tissue‐ and growth‐stage‐specific manner (Tables 6, 7, 8). At 600 ppm CO_2_, certain lignin monomers, such as guaiacyl (G) and syringyl (S), increased in specific tissues, whereas higher CO_2_ levels (1200–1800 ppm) generally reduced these components and the S/G ratio, indicating complex and genotype‐dependent responses. The expression analysis of lignin biosynthesis‐related genes revealed significant transcriptional changes under elevated CO_2_ (Fig. 7). Genes for key enzymes, including phenylalanine ammonia‐lyase (*PAL*), cinnamyl alcohol dehydrogenase (*CAD*), cinnamoyl‐CoA reductase (*CCR*), 4‐coumarate:CoA ligase (*4CL*), caffeic acid O‐methyltransferase (*COMT*) and caffeoyl‐CoA O‐methyltransferase (*CCoAOMT*), exhibited peak expression at 600 ppm CO_2_ across various growth stages. By contrast, their expression was reduced at higher CO_2_ concentrations (1200 ppm and 1800 ppm), particularly in B73 plants. Similar studies in *Populus* species report variable responses, with PAL and CAD activities in *Populus tremula* remaining stable in opposite wood but decreasing in tension wood after 60 days,^57^ whereas *Populus deltoides* showed increased expression at 800–1200 μmol mol^−1^ CO_2_.^58^ These discrepancies likely reflect species‐specific responses, photosynthetic efficiency, carbon allocation, growth stages or experimental conditions. PAL, a key enzyme in phenylpropanoid metabolism, catalyzes the deamination of l‐phenylalanine into *trans*‐cinnamic acid, the initial step of lignin biosynthesis.^59^ Elevated CO_2_ has been reported to stimulate carbon allocation to the phenylpropanoid pathway, particularly enhancing PAL activity.^60^ All of these results demonstrate that elevated CO_2_ modulates lignin biosynthesis in maize through a complex interplay of genotype, tissue type and growth stage. These findings underscore the importance of lignin‐related metabolic pathways in shaping plant structural integrity and stress resilience under changing atmospheric CO_2_ levels. Further research is needed to elucidate the underlying molecular mechanisms and their implications for crop adaptation to future climate scenarios.

## SUMMARY

The present study investigates the effects of elevated CO_2_ concentrations on the growth, biomass accumulation, folate derivatives, starch, sugars and lignin biosynthesis in maize inbred lines C01 and B73. The results reveal significant genotype‐specific responses to elevated CO_2_ levels. Both lines exhibited increased height and biomass at 600 ppm, with growth declining at 1800 ppm, particularly at 70 DAS. Leaf area was highest at 600 ppm for C01 at 40 DAS and for B73 at 70 DAS. Elevated CO_2_ at 600 ppm enhanced starch, soluble sugars and TNC for both genotypes, whereas these components declined at 1800 ppm, especially at 90 DAS. Chlorophyll and carotenoid levels decreased under elevated CO_2_, with C01 showing more pronounced reductions. Folate content peaked at 70 DAS for both genotypes, with B73 showing higher folate levels across all stages at 600 ppm, whereas C01 exhibited reductions at higher CO_2_ levels. Lignin content was inversely proportional to CO_2_ levels, with significant reductions at 1800 ppm, and the composition of lignin monomers varied by genotype, growth stage and CO_2_ concentration. Phloroglucinol staining indicated reduced lignification in response to elevated CO_2_, with C01 showing stronger staining, suggesting inherently higher lignin content. The expression of lignin biosynthesis genes, including *ZmPAL*, *ZmCAD*, *ZmCCR*, *Zm4CL* and *ZmCOMT*, varied between genotypes and CO_2_ levels, indicating genotype‐specific regulation of lignin biosynthesis. Future research should explore the molecular mechanisms driving these genotype‐specific responses, particularly in lignin and folate biosynthesis, as well as investigate the impact of elevated CO_2_ on other biochemical pathways. Long‐term field studies under varying CO_2_ levels are needed to assess how climate change may affect maize productivity and nutritional content. Breeding strategies targeting enhanced growth and stress resilience under elevated CO_2_ should also be prioritized.

## AUTHOR CONTRIBUTIONS

PK, SA, L and ERE were responsible for conceptualization, methodology and writing original draft. PK and RJ were responsible for experiments, formal analysis and visualization. RJ and KMK were responsible for resources, supervision and funding acquisition. All authors have read and approved the final version of the manuscript submitted for publication.

## FUNDING

This research was supported by the Regional Innovation System & Education (RISE) Glocal 30 program through the Daegu RISE Center, funded by the Ministry of Education (MOE) and the Daegu, Republic of Korea (2025‐RISE‐03‐001). This work was carried out with the support of the ‘Cooperative Research Program for Agriculture Science and Technology Development (Project No. RS‐2025‐00512751)’ by the Rural Development Administration, Republic of Korea. We extend our appreciation to Northern Border University, Saudi Arabia, for supporting this work (Project number NBU‐CRP‐2025‐249).

## CONFLICTS OF INTEREST

The authors declare that they have no conflicts of interest.

## Supporting information


**Table S1.** Primer list and gene accession number.

## Data Availability

The data that supports the findings of this study are available in the supplementary material of this article.
