# Supplementary material for: The influence of rising carbon dioxide on maize development: genotypic differences in growth, lignification and folate pathway
Source: J Sci Food Agric. 2025 Oct 16;106(2):1283–98. doi: 10.1002/jsfa.70251 (PMC12701294; doi:10.1002/jsfa.70251)
Supplement: Supplementary file 1 — Table S1. Primer list and gene accession number. [file JSFA-106-1283-s001.docx]

| Gene name | Primer sequence | Accession number |
| --- | --- | --- |
| *ZmPAL* | F- AAGGAGAAGAGGAGGGAGGG  R- GAAGAAAGAGCAACGCCACA | XM_020537583.3 |
| *Zm4CL* | F- CGACATCGAGATCGACAGCA  R- ATGATCACGATGGTGGAGCC | AY566301.1 |
| *ZmCAD* | F- GCCGACTCGCTGGACTACATCA  R- TCTCGTCGATGCTGCCGATGAA | EU973153.1 |
| *ZmCOMT* | F- ATGGCAAGGTCATCGTCGTCG  R- AGGCGTTGGCGTAAATGTAGGT | KF020740.1 |
| *ZmCCR* | F- GCGGAAGCAGCCGTACAAGT  R- CCTGGAGGTTCTTCACCGTGTC | X98083.1 |
| *ZmCCoAOMT* | F- CAAGAGCGACGACCTGTACC  R- GGGCCAAGAAAGAGCCAGAT | XM_023300961.2 |

Table S1. Primer list and gene accession number.
